# Anti-metastatic effect of the TM4SF5-specific peptide vaccine and humanized monoclonal antibody on colon cancer in a mouse lung metastasis model

**DOI:** 10.18632/oncotarget.13005

**Published:** 2016-11-01

**Authors:** Guang Wu, Dongbum Kim, Byoung Kwon Park, Sangkyu Park, Ji-Hee Ha, Te Ha Kim, Avishekh Gautam, Jung Nam Kim, Su In Lee, Han-Bum Park, Yong-Sung Kim, Hyung-Joo Kwon, Younghee Lee

**Affiliations:** ^1^ Center for Medical Science Research, College of Medicine, Hallym University, Chuncheon 24252, Republic of Korea; ^2^ Department of Biochemistry, College of Natural Sciences, Chungbuk National University, Cheongju 28644, Republic of Korea; ^3^ Department of Molecular Science and Technology, Ajou University, Suwon 16499, Republic of Korea; ^4^ Department of Microbiology, College of Medicine, Hallym University, Chuncheon 24252, Republic of Korea

**Keywords:** TM4SF5, peptide vaccine, monoclonal antibody, colon cancer, anti- metastatic effect

## Abstract

Transmembrane 4 superfamily member 5 protein (TM4SF5) is a potential therapeutic target for hepatocellular carcinoma (HCC) and colon cancer. In a previous study, we demonstrated the prophylactic and therapeutic effects of a TM4SF5-specific peptide vaccine and monoclonal antibody in HCC and colon cancer in a mouse model. Here, we designed a cyclic peptide targeting TM4SF5. Cyclic peptide-specific antibodies were produced in mice after immunization with a complex of the peptide, CpG-DNA, and liposomes. Intravenous injection of the CT-26 mouse colon cancer cell line into mice induced tumors in the lung. Immunization with the peptide vaccine improved the survival rate and reduced the growth of lung tumors. We established a monoclonal antibody specific to the cyclic TM4SF5-based peptide and humanized the antibody sequence by complementarity determining region-grafting. The humanized antibody was reactive to the cyclic peptide and TM4SF5 protein. Treatment of CT-26 cells with the humanized antibody reduced cell motility *in vitro*. Furthermore, direct injection of the humanized anti-TM4SF5 antibody *in vivo* reduced growth of lung tumors in mouse metastasis model. Therefore, we conclude that the immunization with the cyclic peptide vaccine and injection of the TM4SF5-specifc humanized antibody have an anti-metastatic effect against colon cancer in mice. Importantly, the humanized antibody may serve as a starting platform for further development and application in clinical settings.

## INTRODUCTION

Metastasis is the most critical factor in cancer-induced fatality. Therefore, understanding the molecular mechanisms of metastasis and finding strategies to suppress metastasis are the most important considerations in anti-cancer therapeutics [1, 2]. Colon cancer is one of the most frequently occurring cancers worldwide [3]. Some populations of colon cancer patients have tumors that metastasize to the liver, lung, and peritoneum [1].

The transmembrane 4 superfamily member 5 protein (TM4SF5) belongs to the tetraspanin family, which is characterized by four hydrophobic transmembrane domains. TM4SF5 is involved in cancers such as hepatocellular carcinoma (HCC) and colon cancer [4–9]. TM4SF5 is known to be involved in epithelial-mesenchymal transition (EMT) and to enhance uncontrolled cell proliferation in HCC [5, 6]. Overexpression of TM4SF5 enhances migration and invasion of HCC, leading to increased lung metastasis [10]. TM4SF5-mediated focal adhesion kinase (FAK) activation seems to contribute to alteration of integrin-mediated cell adhesion and metastasis of HCC [11]. Recently, involvement of TM4SF5 and CD44 in the increase of circulating tumor cells has been suggested in HCC [12, 13]. Therefore, TM4SF5 has been proposed to be a reasonable target in management of HCC metastasis.

Previously, we reported that TM4SF5 can be a therapeutic target of vaccination for HCC and colon cancer [7–9]. We showed that a peptide vaccine composed of liposome-encapsulated CpG-DNA and a B-cell epitope, predicted from the amino acid sequence of TM4SF5, contributed to prevention and therapy of HCC and colon cancer in a mouse model [7–9]. Furthermore, we produced a monoclonal antibody that specifically recognizes human and mouse TM4SF5 and confirmed that the anti-TM4SF5 monoclonal antibody has a therapeutic effect in mouse models for HCC and colon cancer [14, 15]. Moreover, we showed that treating TM4SF5-expressing HCC cells with the anti-TM4SF5 monoclonal antibody induces an increase in E-cadherin expression and decreases the migration capacity of the cells [14]. We also confirmed that E-cadherin and β-catenin expression was increased by anti-TM4SF5 monoclonal antibody treatment in human colon cancer cells [15]. Therefore, it is highly possible that a TM4SF5-specific peptide vaccine and an anti-TM4SF5 monoclonal antibody can be valuable agents in clinical management of HCC and colon cancer metastases. However, the off-rate of the anti-TM4SF5 monoclonal antibody after target binding is high, therefore it is necessary to isolate antibody with higher affinity for future application. Humanization and evaluation of the obtained monoclonal antibody is also required.

In this study, we used a cyclic peptide that mimics a structural motif of TM4SF5 as an antigen and successfully isolated the monoclonal antibody that recognizes TM4SF5 protein with a low off-rate. Furthermore, we produced a humanized antibody and evaluated its reactivity *in vitro* and *in vivo*. Importantly, we found that the cyclic peptide vaccine and the humanized anti-TM4SF5 antibody suppressed the formation and growth of lung metastases, which were established by intravenous injection of colon cancer cells in a mouse metastasis model.

## RESULTS

### Immunization with the TM4SF5 peptide vaccine and production of antibodies specific to the cyclic peptide of TM4SF5

**Figure 1:**
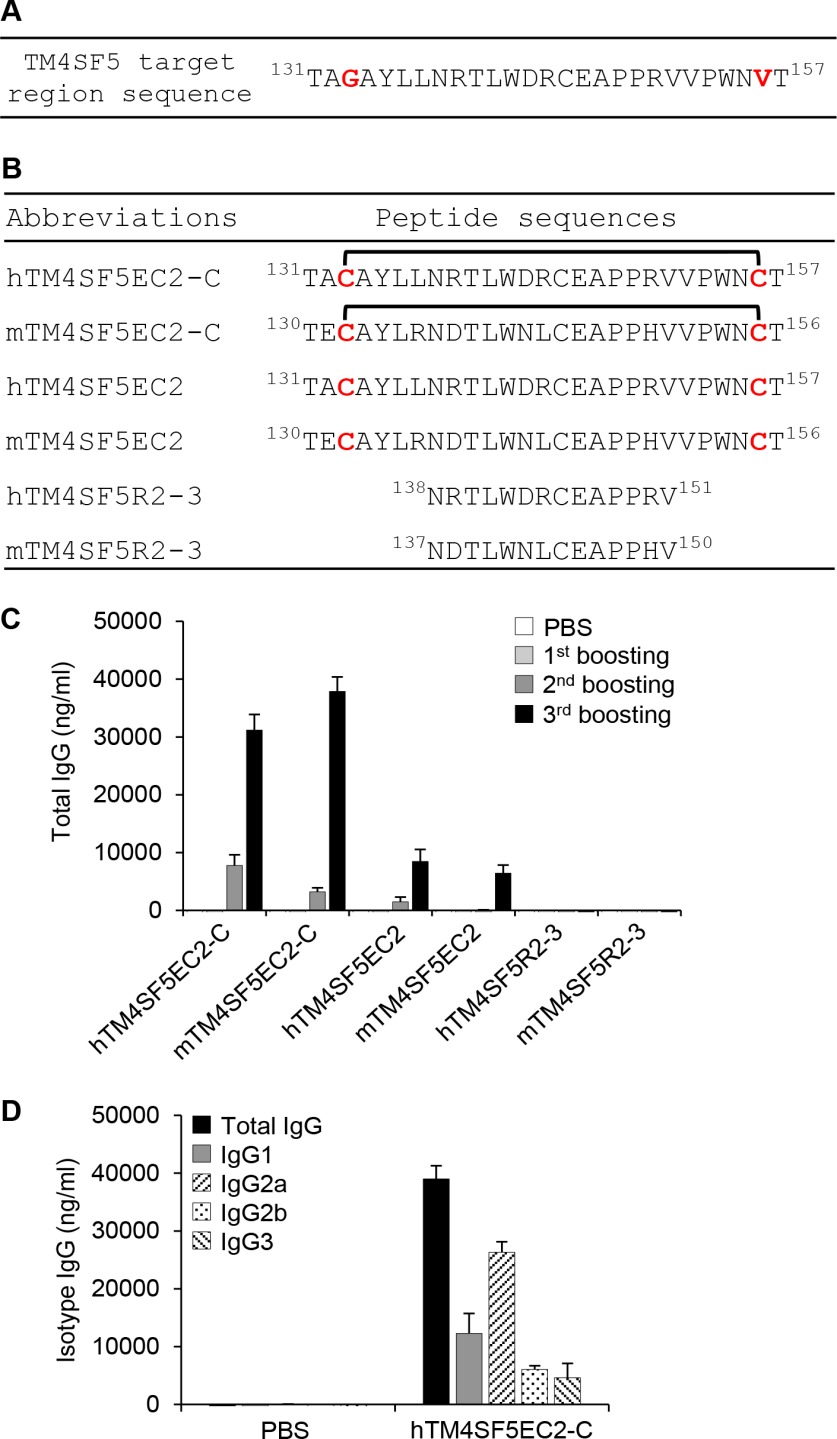
Induction of antibodies in mice immunized with a TM4SF5-based cyclic peptide vaccine (**A**) The sequence of the TM4SF5 target region. (**B**) The sequences of the peptides used in this study. hTM4SF5EC2-C and mTM4SF5EC2-C represent the cyclic peptides in which hTM4SF5EC2 and mTM4SF5EC2 are self-linked by a disulfide bond. (**C**) BALB/c mice were injected with PBS or hTM4SF5EC2-C peptide, along with Lipoplex(O) complex, three times at 10 day intervals (*n* = 5 per group). The titers and the reactivity of the antibodies in the serum samples were measured by ELISA using the indicated peptides. (**D**) The isotypes of the antibodies reactive to the hTM4SF5EC2-C peptide were characterized by ELISA for isotyping.

We designed a cyclic peptide that could potentially mimic the structural motif of TM4SF5 extracellular domain 2 (EC2) to obtain antibodies that recognize TM4SF5 structural epitopes and retain tight binding. We replaced glycine 133 and valine 156 with cysteine residues to construct a mutant peptide, hTM4SF5EC2 (Figure 1A and 1B). Through chemical modification of the peptide, we produced a cyclic peptide hTM4SF5EC2-C with a disulfide bond between the cysteine residues. We immunized mice with liposome complexes, including the hTM4SF5EC2-C peptide and CpG-DNA co-encapsulated with liposome [Lipoplex(O)], and confirmed robust production of antibodies that recognize the hTM4SF5EC2-C cyclic peptide after the third boosting immunization (Figure 1C). The antibody cross-reacted with the corresponding mouse cyclic peptide (mTM4SF5EC2-C), and its reactivity to the linear peptides hTM4SF5EC2 and mTM4SF5EC2 was much lower than that to the cyclic peptides. The antibodies did not recognize hTM4SF5R2-3, which was used as an antigen in a previous study [7], or the corresponding mouse epitope mTM4SF5R2-3, suggesting that the produced antibody recognizes a conformational epitope. As shown in Figure 1D, the antibodies produced were primarily of the IgG2a isotype.

### Immunization with the TM4SF5 peptide vaccine inhibits growth of colon tumors in a mouse lung metastasis model

**Figure 2:**
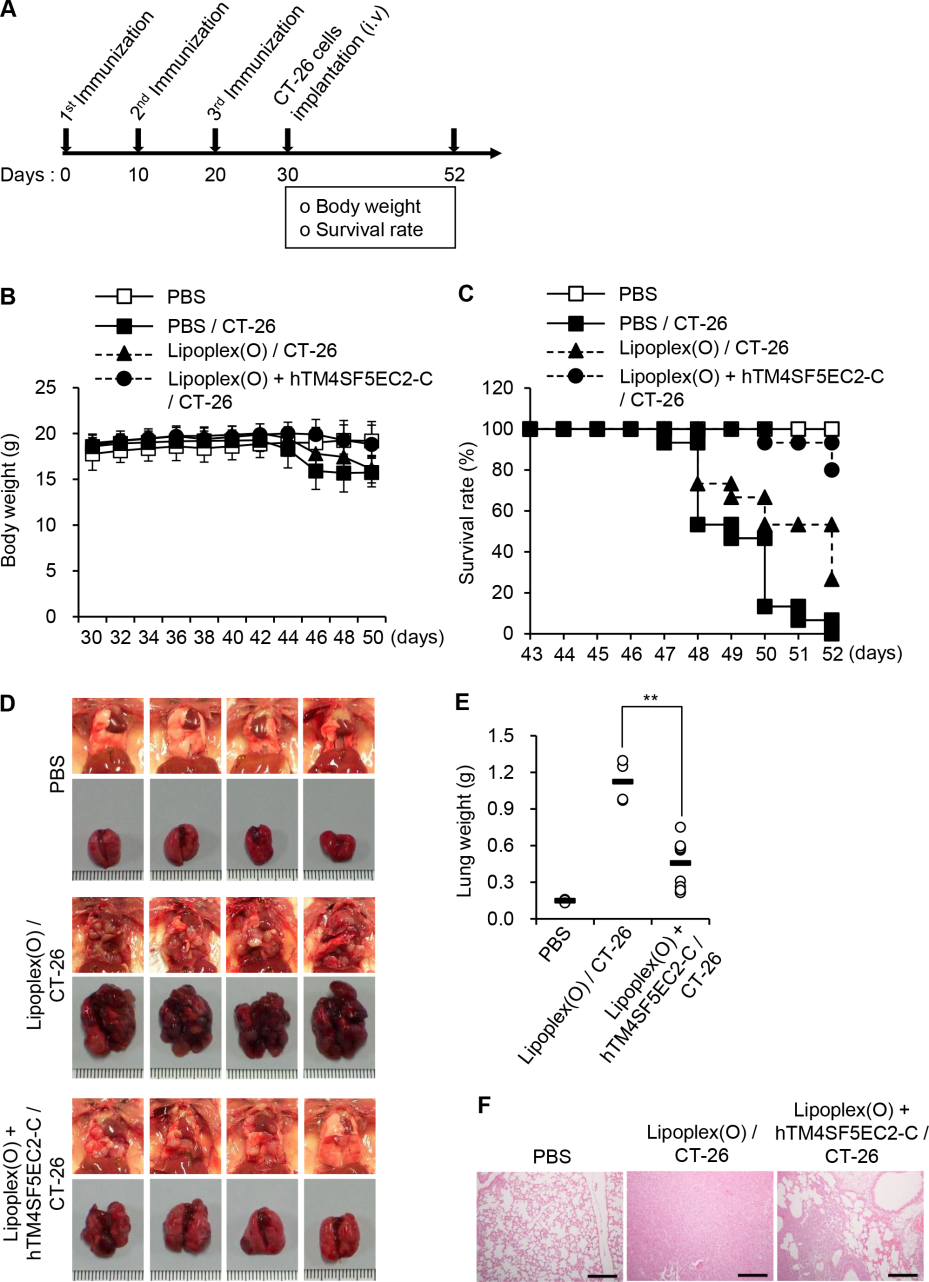
Inhibition of lung metastasis by immunization with TM4SF5 cyclic peptide vaccine in a mouse model of colon cancer BALB/c mice were injected with PBS, Lipoplex(O), or the complex of hTM4SF5EC2-C peptide and Lipoplex(O) at 10 day intervals (PBS controls, *n* = 8; colon cancer cell-injected group, *n* = 15). A metastasis model was established by intravenous implantation of CT-26 cells in the treated mice, and the body weight and survival rate of the mice was monitored. (**A**) Experimental schedule. (**B**) Body weights were measured every other day for 20 days after CT-26 cell implantation. (**C**) Survival of the immunized mice after CT-26 cell implantation. (**D**) Macroscopic appearance of lungs examined at day 52. (**E**) Lung weight of the mice at day 52. **P < 0.01. (**F**) Histological examination of the lung tissues. Scale bars, 100 μm.

**Figure 3:**
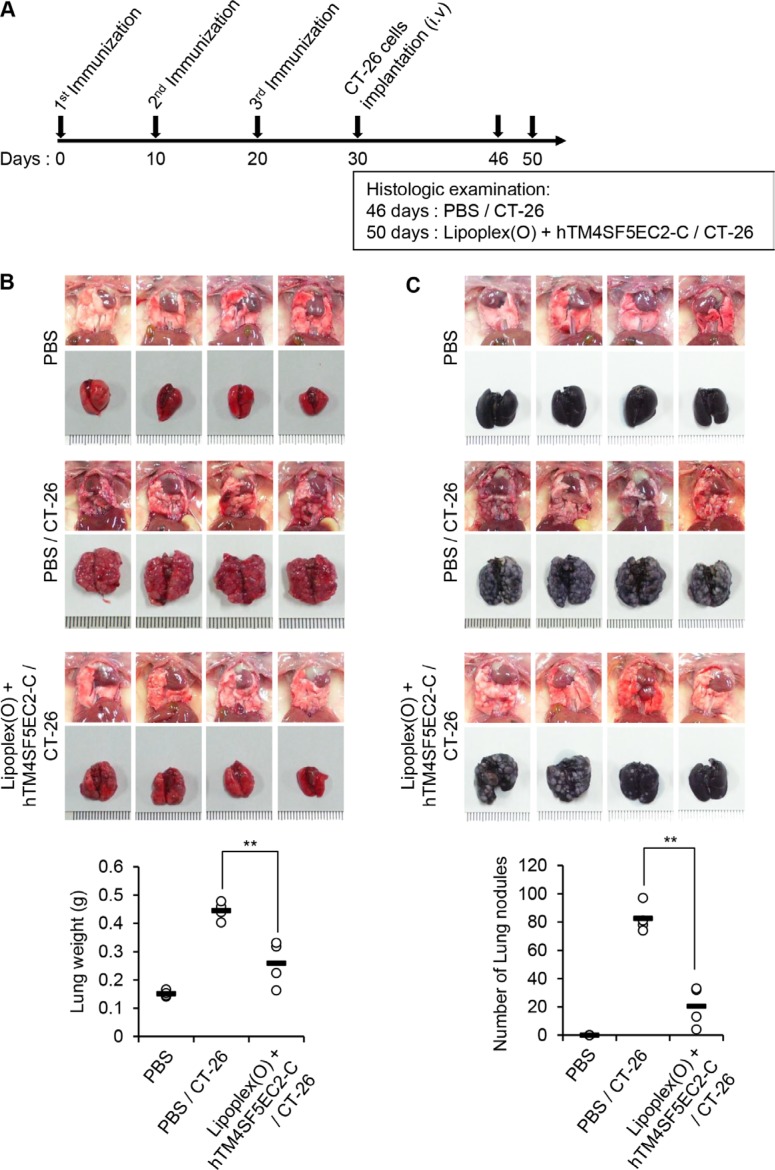
Reduction of lung nodule numbers by immunization with a TM4SF5 peptide vaccine in a mouse model of colon cancer BALB/c mice were injected with PBS or the hTM4SF5EC2-C peptide and Lipoplex(O) complex at 10 day intervals (*n* = 8 per group). The metastasis model was established as described in Figure 2, and the tumor growth was monitored until day 46 or 50. (**A**) Experimental schedule. (**B**) Macroscopic appearance of lungs and lung weight examined at day 46 (CT-26 group) and day 50 (PBS control group, Lipoplex(O) + hTM4SF5EC2-C peptide/CT-26 group; *n* = 4 per group). (**C**) Number of lung nodules at day 46 (CT-26 group) and day 50 (PBS control group, Lipoplex(O) + hTM4SF5EC2-C peptide/CT-26 group; *n* = 4 per group). **P < 0.01.

To evaluate the significance of TM4SF5 as a target in colon cancer metastasis control in mice, we first immunized BALB/c mice with the TM4SF5 peptide vaccine composed of the cyclic TM4SF5 peptide (hTM4SF5EC2-C) and Lipoplex(O). Then, we assessed the effect of the TM4SF5 peptide vaccine on the growth of lung tumors induced by intravenous injection of CT-26 colon cancer cells (Figure 2A). The mice injected with CT-26 cells underwent loss of body weight approximately 12 days after injection of the cells. However, the mice immunized with the TM4SF5 peptide vaccine showed a body weight pattern similar to that of the untreated control mice (Figure 2B). Compared with mice that received the phosphate-buffered saline (PBS) control, survival of the mice that received the TM4SF5 peptide vaccine was greatly enhanced (Figure 2C; 80% versus 0% at day 52). Immunization with the CpG-DNA-liposome complex [Lipoplex(O)] without the peptide induced a partial protective effect that may be due to the non-specific immunostimulatory effect (27% at day 52). Using tumor volume and weight as indicators, we observed that immunization with the TM4SF5 peptide vaccine reduced the progression of lung metastatic tumors compared to treatment with PBS or Lipoplex(O) controls (Figure 2D–2E). Histological examination showed that lung tissue of the peptide-vaccinated mice was morphologically similar to that of normal mice (Figure 2F). To confirm the anti-metastatic effect of the TM4SF5 peptide vaccine, we repeated the above trial and monitored metastatic nodules in the lung as an indicator of metastasis progression. Immunization with the TM4SF5 peptide vaccine significantly reduced the number of lung nodules, compared with that in the PBS control (Figure 3). Taken together, these results suggest that immunization with the TM4SF5 peptide vaccine can attenuate lung metastasis of colon tumors in the mouse model.

### Isolation and characterization of a monoclonal antibody specific to TM4SF5 cyclic peptide

**Figure 4:**
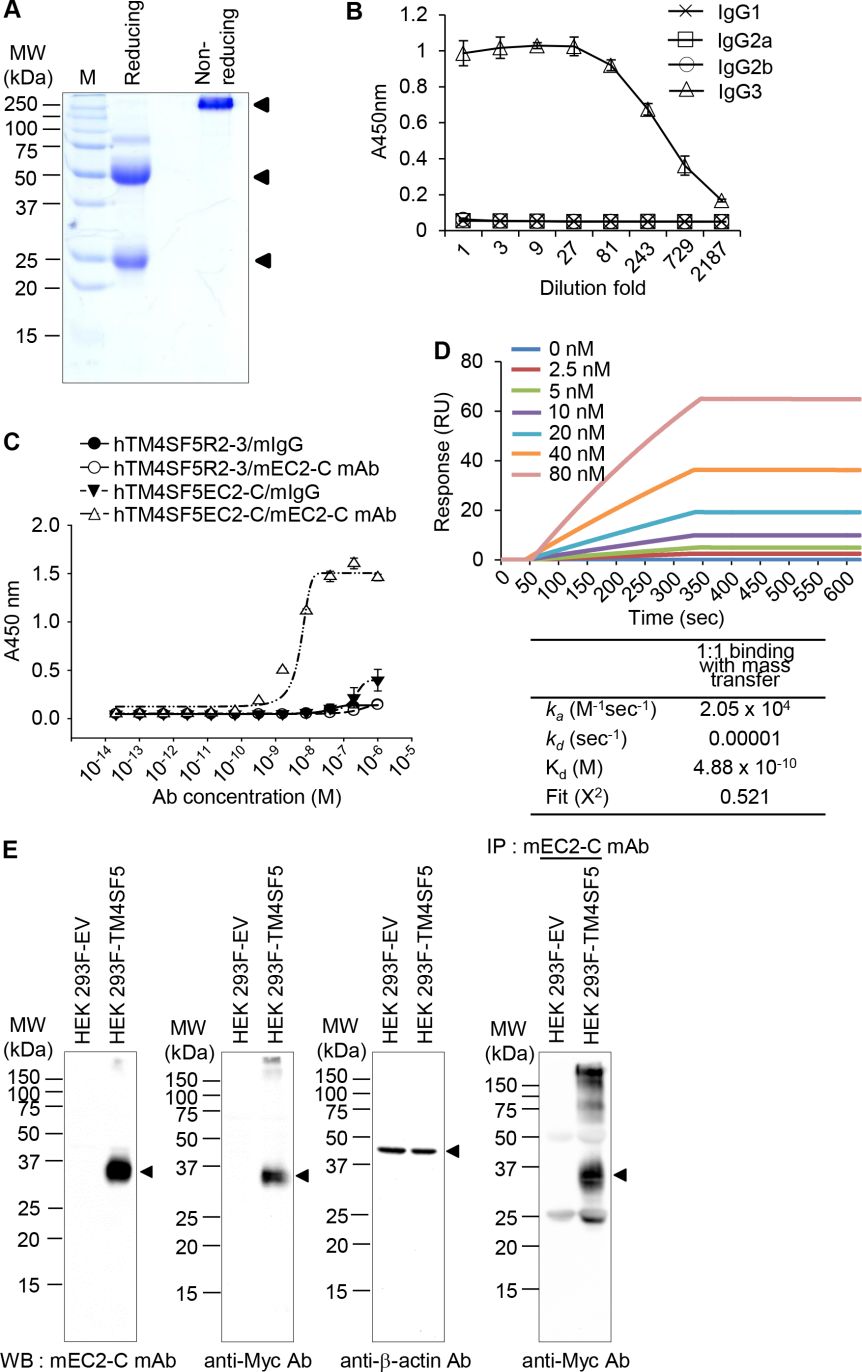
Purification and characterization of the mouse anti-TM4SF5 monoclonal antibody that recognizes the TM4SF5 cyclic peptide (**A**) Purified anti-TM4SF5 monoclonal antibody was evaluated by SDS-PAGE and Coomassie blue staining. (**B**) The titration curve was obtained using purified monoclonal antibody to determine the isotype. (**C**) Determination of monoclonal antibody binding affinity for the cyclic peptide hTM4SF5EC2-C using ELISA. (**D**) Determination of binding affinity for the cyclic peptide hTM4SF5EC2-C using SPR system. Biotinylated peptides were immobilized on a streptavidin chip, and increasing amounts of antibody were applied. Kinetic parameters are shown under the sensorgrams. (**E**) Whole cell protein lysates from HEK 293F-EV (empty vector) and HEK 293F-TM4SF5 cells were analyzed by western blotting with anti-TM4SF5 monoclonal (mEC2-C), anti-Myc, or anti-β-actin antibodies. Whole cell protein lysates from HEK 293F-EV and HEK 293F-TM4SF5 cells were immunoprecipitated with mEC2-C and analyzed by western blotting with the anti-Myc antibody. These results are representative of at least three independent experiments.

We screened hybridoma cell lines and successfully isolated a monoclonal antibody that recognizes the hTM4SF5EC2-C peptide. We purified monoclonal antibodies from ascites (Figure 4A) and found that the prepared monoclonal antibody was of the IgG3-isotype (Figure 4B). We named the monoclonal antibody mEC2-C and confirmed its specific binding to the cyclic peptide hTM4SF5EC2-C using enzyme-linked immunosorbent assay (ELISA; Figure 4C). The binding affinity of the antibody to hTM4SF5EC2-C peptide was measured by surface plasmon resonance (SPR) analysis as shown in Figure 4D. The equilibrium dissociation constant (K_d_) of the antibody was 0.48 nM, which is about 5-fold lower than the value of the previously-reported antibody (clone #2D4-18, obtained using a linear epitope hTM4SF5R2-3 as an antigen; 2.74 nM) [14]. The off-rate (*k*_off_) of the antibody was 10^−5^/sec, which is much slower than the clone #2D4-18 (5.99 × 10^−2^/sec). Therefore, we conclude that the antibody generated using the hTM4SF5EC2-C peptide may be more useful for clinical application. We performed western blotting and immunoprecipitation analyses using HEK 293F control cells and HEK293F-TM4SF5 cells (overexpressing TM4SF5), and confirmed that the antibody recognizes Myc-tagged recombinant TM4SF5 protein (Figure 4E).

### Production and characterization of humanized monoclonal antibody

**Figure 5:**
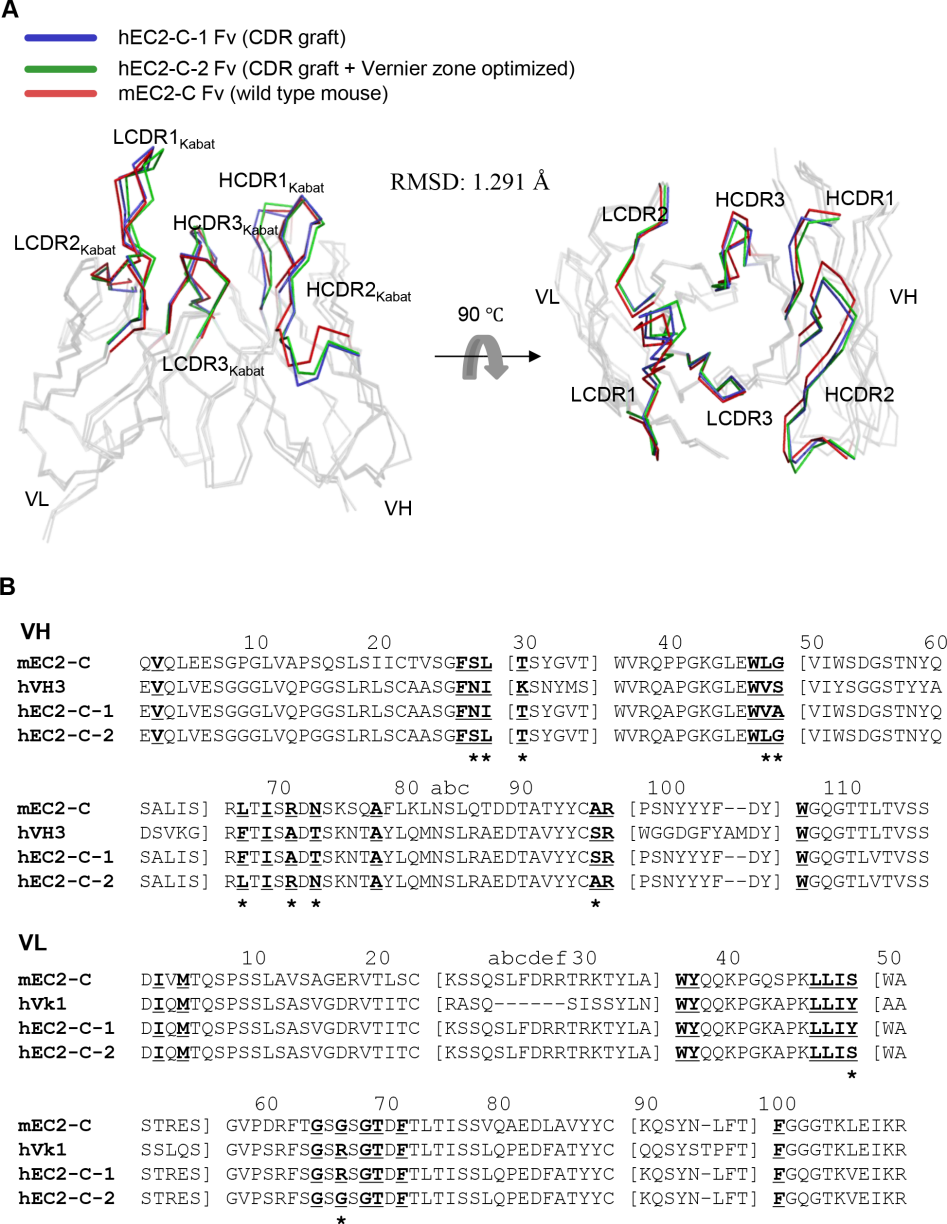
Sequence and structural analysis for humanization of mEC2-C monoclonal antibody (**A**) Superposition of homology-modeled antibody Fv structures. The alpha carbon traces of VH and VL are displayed with highlights of the CDRs of each antibody in the indicated color code. The images were generated using PyMol software (DeLano Scientific LLC). (**B**) Amino acid sequence alignment using VH and VL domains of mEC2-C and hEC2-C monoclonal antibodies. The sequence of Herceptin Fc used as the acceptor human framework for mEC2-C humanization is also shown as hVH3 and hVk1 for comparison. CDR residues are indicated by a square bracket ([ ]). The Vernier zone residues in the β-sheet framework closely underlying CDRs are underlined and bolded. The 11 mouse Vernier zone residues retained in the humanized hEC2-C-2 antibody are indicated by asterisk (*). The Kabat numbering system was used for definition of framework residues and CDRs.

For application of monoclonal antibodies in clinical settings, the antibodies have to be humanized to reduce immunogenicity in humans [16]. Therefore, we analyzed the immunoglobulin variable domain sequence of the obtained monoclonal antibody mEC2-C using the IgBLAST program [17] and found that the variable domain subtype belongs to mouse VH2-Vk8. For humanization of mEC2-C monoclonal antibody, we chose VH3-Vk1 framework because these frameworks are the most commonly observed in human germ line repertoire [18] and have also been successfully used in the humanized antibodies that are currently available commercially [19]. We engrafted the complementarity determining regions (CDRs) and some framework sequences to the human VH3-Vk1 framework (Herceptin framework, in this case) using established procedures [20]. The structures of the variable regions from mEC2-2 and the humanized monoclonal antibody (hEC2-C-2) were modeled and compared, and the data show that although the structures do not overlap completely, they are very similar showing a root mean squared deviation (RMSD) of 1.291 Å for the backbone Cα atoms (Figure 5).

**Figure 6:**
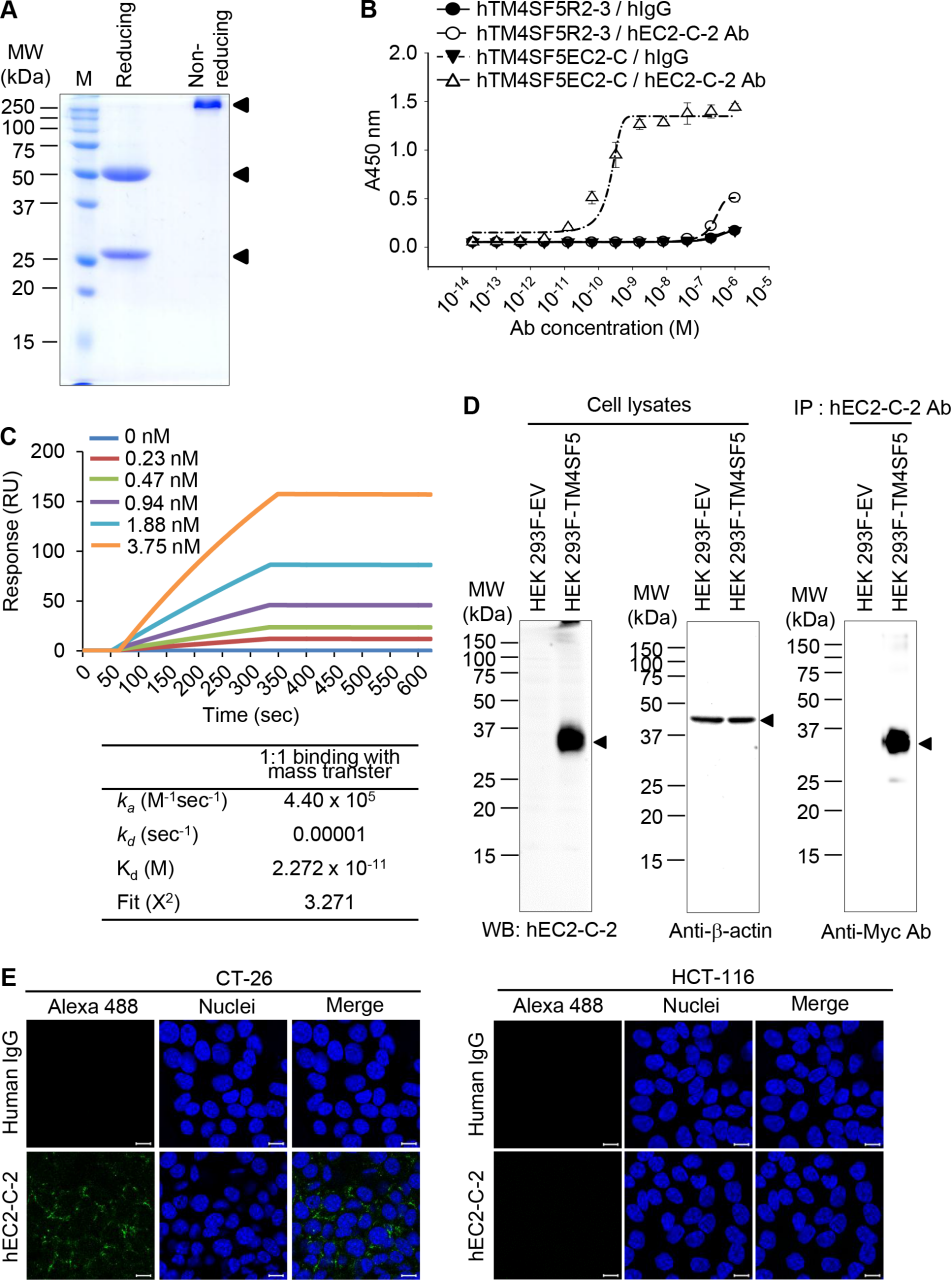
Purification and characterization of humanized anti-TM4SF5 antibody that recognizes the TM4SF5 cyclic peptide (**A**) Purified humanized anti-TM4SF5 antibody was evaluated by SDS-PAGE and Coomassie blue staining. (**B**) Binding affinity of the humanized antibody for the cyclic peptide hTM4SF5EC2-C was analyzed using ELISA. (**C**) Binding affinity for the cyclic peptide was analyzed using SPR system. (**D**) Whole cell protein lysates from HEK 293F-EV and HEK 293F-TM4SF5 cells were analyzed by western blotting with humanized anti-TM4SF5 antibody (hEC2-C-2) or anti-β-actin antibody. Whole cell protein lysates from HEK 293F-EV and HEK 293F-TM4SF5 cells were immunoprecipitated with hEC2-C-2 antibody and analyzed by western blotting with the anti-Myc antibody. (**E**) CT-26 and HCT-116 cells were analyzed by confocal microscopy using normal human IgG and hEC2-C-2 antibody. Scale bars, 20 μm.These results are representative of at least three independent experiments.

We produced recombinant humanized monoclonal antibody (hEC2-C-2) in HEK 293F cells (Figure 6A) and evaluated its reactivity (Figure 6B–6E). The humanized antibody was specifically reactive to the cyclic peptide hTM4SF5EC2-C, but not to hTM4SF5R2-3, as assessed using ELISA (Figure 6B). The equilibrium dissociation constant (K_d_) of the antibody was approximately 22.7 pM, which is about 20-fold lower than the original mouse monoclonal antibody mEC2-C (Figure 6C). The humanized antibody can detect TM4SF5 protein in HEK 293F cells that overexpress TM4SF5, as evaluated using western blotting and immunoprecipitation analyses (Figure 6D). The confocal image analysis showed that the humanized antibody recognizes TM4SF5 protein in CT-26 cells, which were previously confirmed to express TM4SF5 (Figure 6E). There was no signal detected in HCT-116 cells, a TM4SF5-negative cell line. Hence, we can conclude that the humanized antibody is fully reactive to TM4SF5 protein and has higher affinity compared with the original monoclonal antibody.

### Effect of the humanized anti-TM4SF5 antibody on migration and β-catenin expression in colon cancer cells

**Figure 7:**
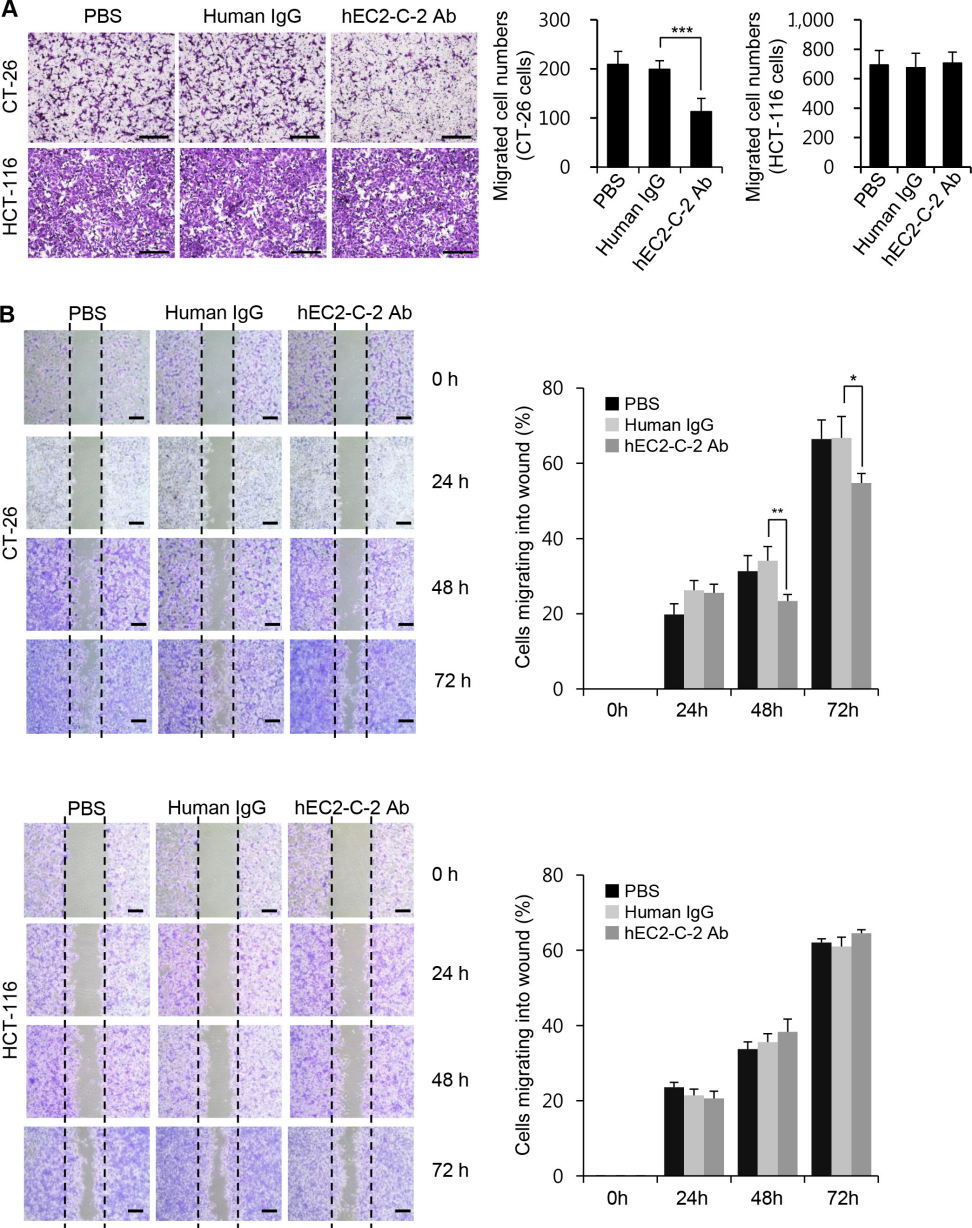
Effects of the humanized anti-TM4SF5 monoclonal antibody on the migration of colon cancer cells (**A**) Migration assay. Scale bars, 100 μm. (**B**) Wound-healing assay. Scale bars, 300 μm. Migration properties of CT-26 and HCT-116 cells were compared after treatment with PBS, normal human IgG, or hEC2-C-2 antibody. These results are representative of three independent experiments. Results are presented as mean + standard deviation. *P < 0.05, **P < 0.01, ***P < 0.001.

TM4SF5 activates integrin-mediated signaling pathways that are pivotal for cell migration/invasion and tumor cell metastasis [10–14]. Therefore, we evaluated the influence of the humanized anti-TM4SF5 antibody *in vitro* on cell migration using CT-26 and HCT-116 cells. The addition of the humanized anti-TM4SF5 antibody, but not PBS or human IgG control, inhibited the migration of CT-26 cells (Figure 7A). In contrast, the antibody had little effect on the migration of HCT-116 cells, which do not express TM4SF5. In addition, we performed wound-healing assays *in vitro*. The migration of CT-26 cells into the wounded area was significantly inhibited by the humanized anti-TM4SF5 antibody, compared to the PBS or human IgG control (Figure 7B). However, there was no difference in the wound healing capacity in HCT-116 cells treated with PBS, human IgG control, or the humanized anti-TM4SF5 antibody.

**Figure 8:**
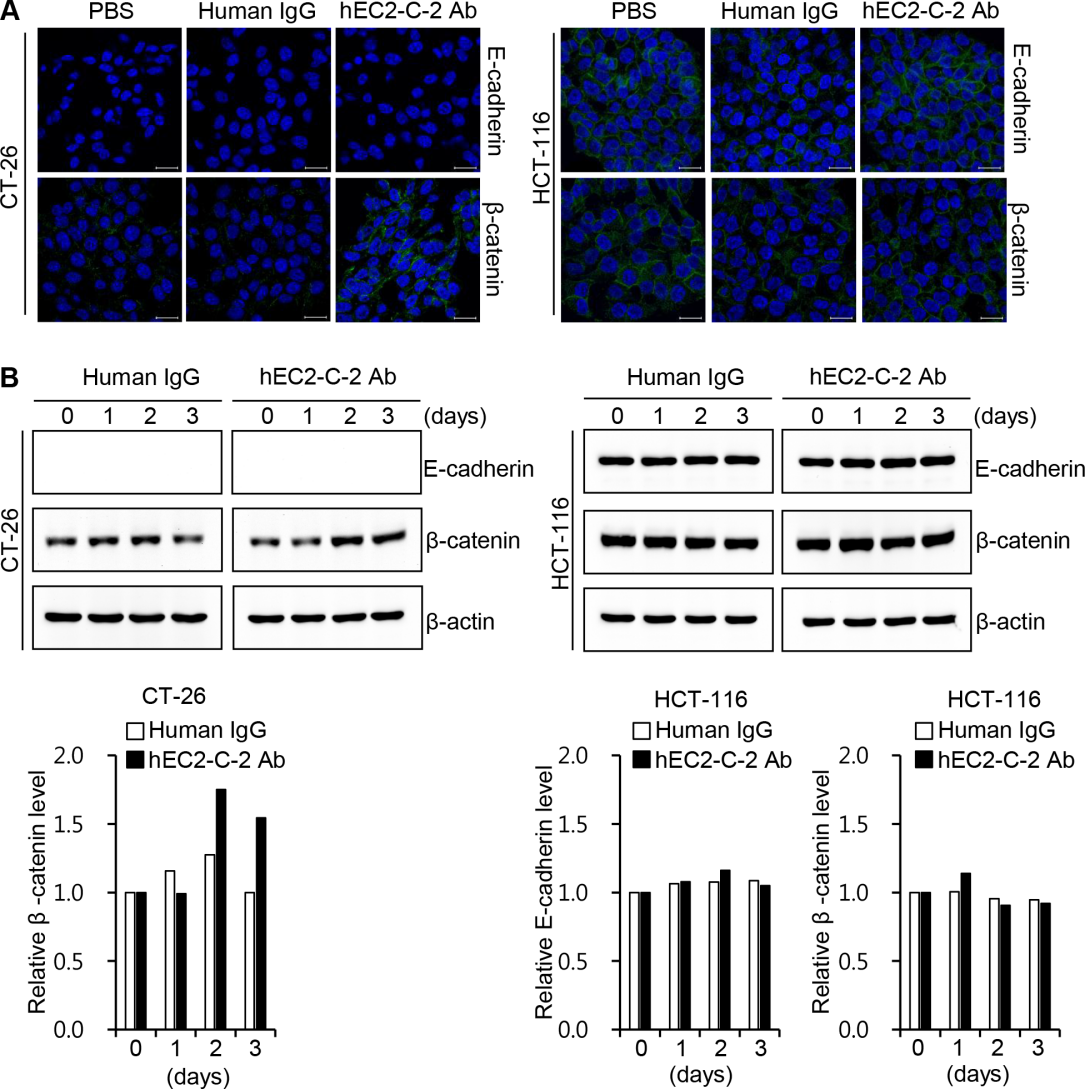
Effect of the humanized anti-TM4SF5 antibody on the expression of adhesion molecules in colon cancer cells CT-26 and HCT-116 cells were treated with PBS, normal human IgG, or hEC2-C-2 antibody. Expression of E-cadherin and β-catenin was determined by western blotting with anti-E-cadherin and anti-β-catenin antibodies. (**A**) Confocal microscopy images after 3 h of treatment with DyLight 488-labeled human IgG control or humanized anti-TM4SF5 antibody (hEC2-C-2 Ab; 10 μg/mL). Scale bars, 20 μm. (**B**) Western blot analysis. β-actin was used as an internal control. The band intensities were measured and quantitative changes are shown on the graph. These results are representative of three independent experiments.

To further characterize the effects of the humanized anti-TM4SF5 antibody on cell-cell interactions, we monitored the expression of E-cadherin and β-catenin, proteins that play a critical role in modulating cell-cell junctions, in CT-26 and HCT-116 cells (Figure 8). The confocal imaging data revealed that β-catenin expression was specifically increased in response to the humanized anti-TM4SF5 antibody in CT-26 cells (Figure 8A). E-cadherin expression was not detectable in CT-26 cells irrespective of the treatment, suggesting that basal expression of E-cadherin is very low in CT-26 cells. In contrast, there was no change in E-cadherin and β-catenin expression after treatment with the humanized anti-TM4SF5 antibody in HCT-116 cells. Western blot analysis data were consistent with those of the confocal microscopy analysis (Figure 8B). Therefore, it is likely that the humanized anti-TM4SF5 antibody increases cell-cell interactions and thereby reduces the migration capability in TM4SF5-expressing cancer cells.

### The humanized anti-TM4SF5 antibody inhibits growth of colon tumors in a mouse lung metastasis model

**Figure 9:**
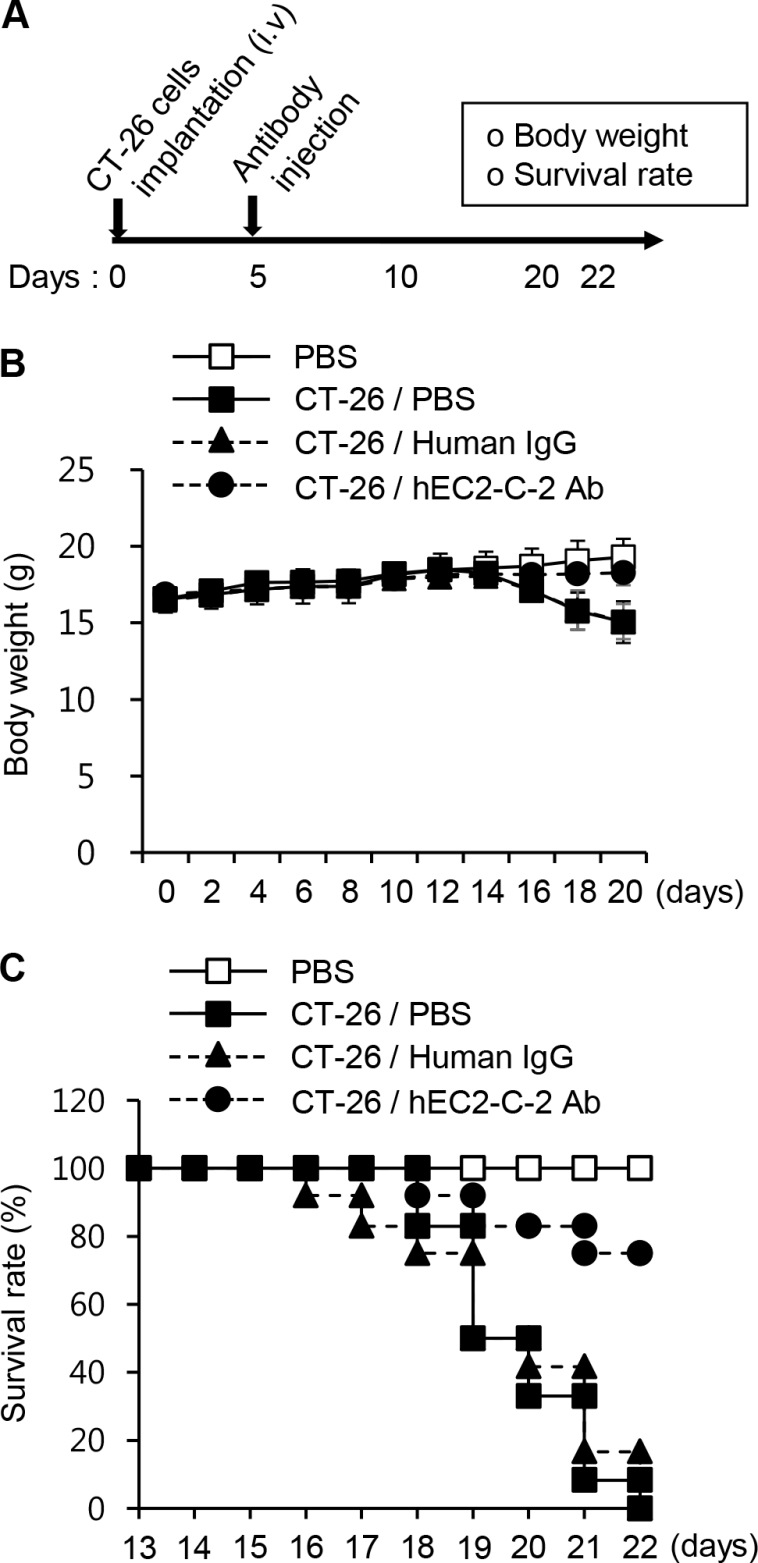
Survival rate in the lung metastasis mouse model CT-26 cells were intravenously injected into BALB/c mice. On day 5, PBS, human IgG, or hEC2-C-2 antibody was intravenously injected into the mice, and tumor growth was monitored after 22 days (PBS controls, *n* = 8; colon cancer cell-injected group, *n* = 12 each). (**A**) Experimental schedule. (**B**) Body weights were measured every other day for 20 days. (**C**) Survival of the humanized anti-TM4SF5 antibody-injected mice after implantation with CT-26 cells.

Because immunization of the mice with the TM4SF5 peptide vaccine reduced growth of lung tumor tissues developed by *i.v.* injection of CT-26 cells, it can be postulated that TM4SF5-specific antibodies induced by immunization directly contribute to the anti-metastatic effects. Therefore, we investigated the effect of the humanized anti-TM4SF5 antibody. First we assessed the effect of the humanized anti-TM4SF5 antibody on body weight and survival of mice in the lung metastasis model (the experimental schedule is shown in Figure 9A). Five days after intravenous injection of CT-26 cells, we intravenously injected normal IgG or the humanized anti-TM4SF5 antibody. The body weight of the control mice was decreased about 16 days after injection with CT-26 cells. However, the mice injected with the humanized anti-TM4SF5 antibody showed body weights similar to those of the untreated control mice (Figure 9B). Survival of the mice was significantly enhanced by the humanized anti-TM4SF5 antibody, compared to those injected with human IgG control (Figure 9C; 75% versus 0%).

**Figure 10:**
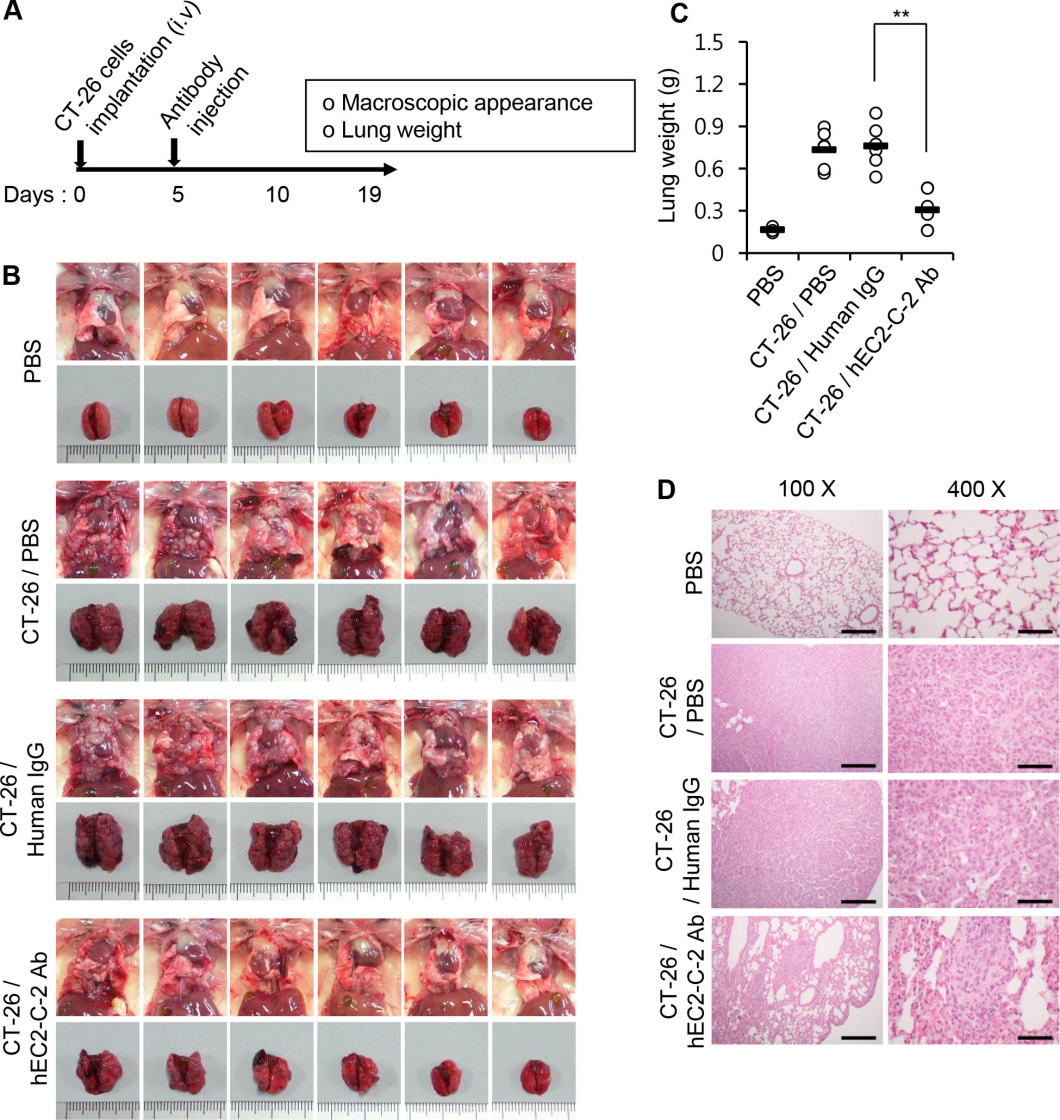
Inhibition of lung metastasis by the humanized anti-TM4SF5 monoclonal antibody CT-26 cells were intravenously injected into BALB/c mice. On day 5, PBS, human IgG, or hEC2-C-2 antibody was intravenously injected into the mice, and tumor growth was monitored after 19 days (PBS controls, *n* = 8; colon cancer cell-injected group, *n* = 12 each). (**A**) Experimental schedule. (**B**) Macroscopic appearance of the lung. (**C**) Weight of the mouse lungs. **P < 0.01. (**D**) Histological examination of the lung tissues. Scale bars: 100X, 100 μm; 400X, 25 μm.

Next, we assessed the formation and progression of lung metastasis in the same experimental model by monitoring the gross appearance of the lungs, lung weight, and histological characteristics of lungs. The formation and growth of lung-metastasized tumors in mice injected with the humanized anti-TM4SF5 antibody was significantly reduced compared with that in PBS-injected control mice (Figure 10). The humanized anti-TM4SF5 antibody reduced the growth of lung metastatic tumors, as measured by changes in tumor volume and weight, compared to that in human IgG-injected mice (Figure 10B–10D). Based on these results, we conclude that the humanized anti-TM4SF5 monoclonal antibody can attenuate lung metastasis of colon tumors in a mouse model.

## DISCUSSION

Metastasis in cancer patients is associated with poor prognosis and death. Therefore, many investigators are trying to find strategies to suppress tumor growth as well as tumor metastasis. Here, we isolated a novel monoclonal antibody targeting a structural epitope of TM4SF5, a protein that induces EMT, proliferation, and metastasis in cancer, and evaluated TM4SF5 as a target of immunotherapy to suppress metastasis of colon cancer in a mouse model.

For application of antibodies as therapeutic reagents in the clinical setting, the antibodies need to have several properties, including a slow off-rate (*k*_off_) after target-binding and low immunogenicity in humans [16, 21, 22]. The antibodies we obtained from a previous study had a high off-rate, suggesting that the antibodies were reactive to TM4SF5 peptide but with insufficient affinity [14]. We postulated that the lower affinity is a consequence of the use of a linear epitope of TM4SF5 as an antigen. Therefore, in this study, we applied a new strategy using a cyclic peptide mimicking a structural motif in the extracellular domain of TM4SF5 as an antigen.

In fact, the advantages of cyclic peptides over linear peptides in cancer management have been shown previously [23–25]. Cyclic peptides named mimotopes were generated and proved to be more effective than linear epitopes in establishment of anti-ErbB-2 monoclonal antibodies [23]. Cyclic peptides, of which the structure is similar to paclitaxel, efficaciously induced apoptosis in many cancer cell lines [24]. Cyclic peptides blocking intracellular protein-protein interaction of Ras signaling components were also suggested as a new anti-cancer agent [25]. Investigators generally make cyclic peptides using disulfide bridges between near cysteine residues. Recently, investigators improved the cyclization efficiency by C-terminal modification and established thioether analogues of disulfide-bridged cyclic peptides, and demonstrated anti-cancer effects of the cyclic peptides [26, 27].

In the first step to try a new approach, we immunized mice with a cyclic peptide vaccine, found that a cyclic peptide-specific antibody was produced, and that tumor formation in the lung was markedly reduced after intravenous injection of CT-26 colon cancer cells. Next, we further isolated monoclonal antibody clones that recognize the cyclic peptide of TM4SF5 and successfully obtained a clone, mEC2-C, with a low off-rate. For future applications, we produced a humanized antibody and evaluated its reactivity.

We previously confirmed that treatment with the anti-TM4SF5 monoclonal antibody reduced the growth of human and mouse colon cancer cells [15]. Here, we found that the treatment of colon cancer cells with the humanized anti-TM4SF5 antibody significantly reduces their migration capability, based on the results from migration and wound healing assays. EMT and migration are necessary for tumors to escape the original location and for circulating tumor cells to penetrate blood vessels in a new location. In fact, treatment with the humanized anti-TM4SF5 antibody induced the expression of β-catenin in TM4SF5-expressing cells, suggesting that cell-cell interaction was reinforced by interruption of TM4SF5 function. The injection of the humanized anti-TM4SF5 antibody significantly suppressed metastatic tumor growth in the lung suggesting that the antibody suppressed re-localization and growth of colon cancer cells in lung tissue.

Considering that the anti-TM4SF5 antibody suppresses tumor growth in HCC and colon cancer models established by subcutaneous cancer cell injection [14, 15], the anti-TM4SF5 antibody might perform a dual anti-cancer function of suppressing the growth of primary tumors, as well as metastasis of the tumors, to other body sites including lung. Xenograft experiments using patient-derived tumor cells [28, 29] have to be performed to evaluate the efficacy of the humanized TM4SF5 antibody for future clinical applications. In addition, the physical properties (solubility and stability), toxicity, immunogenicity, and metabolic data [30, 31] for the humanized antibody have to be evaluated. We will address these issues and the optimization of the humanized antibody for better efficacy in further studies.

## MATERIALS AND METHODS

### Synthesis of CpG-DNA and a cyclic peptide of hTM4SF5EC2

A natural CpG-DNA, MB-ODN 4531(O), comprising 20 bases with three CpG motifs [7] was provided by Samchully Pharm. We designed a cyclic peptide as a potential structural mimic of the extracellular domain 2 of human TM4SF5 (hTM4SF5EC2-C; Figure 1B) and purchased the chemically-synthesized cyclic peptide and control peptides from Peptron.

### Preparation of cyclic peptide epitope and CpG-DNA co-encapsulated with liposome complexes as a peptide vaccine targeting TM4SF5

Liposome complexes consisting of hTM4SF5EC2-C and CpG-DNA co-encapsulated with phosphatidyl-β-oleoyl-γ-palmitoyl ethanolamine (DOPE): cholesterol hemisuccinate (CHEMS) [Lipoplex(O)] were prepared as reported previously [7]. Liposome complexes included MB-ODN 4531(O) (50 μg), peptide (50 μg), and DOPE:CHEMS mixture (at a 1:1 ratio).

### Animals

Female BALB/c mice (four-week-old) were purchased from Nara Biotech, Inc. The mice were maintained under specific pathogen-free conditions at 20–25°C, 32–37% humidity. All procedures for animal experiments were performed according to the Guide for the Care and Use of Laboratory Animals of the National Veterinary Research and Quarantine Service of Korea with the approval of the Institutional Animal Care and Use Committee of Hallym University (Permit Number: Hallym 2015–55, Hallym 2015–56). Mice were sacrificed under isoflurane anesthesia and all efforts were made to minimize suffering.

### ELISA

BALB/c mice were injected intraperitoneally with the TM4SF5 peptide vaccine every 10 days, three times [32]. Control mice were injected with PBS. Mouse sera were obtained by orbital vein bleeding before each intraperitoneal injection and 10 days after final injection. The 96-well immunoplates were coated with 5 μg/mL of each TM4SF5 peptide (described in Figure 1B). Each peptide-specific total IgG amount was measured by ELISA as previously described [9]. To measure the IgG isotype, 96-well immunoplates were coated with the hTM4SF5EC2-C peptide, incubated first with sera, and then with horseradish peroxidase-conjugated anti-mouse IgG (each isotype) antibody (BD Biosciences).

### Cell culture

The HCT-116 human colon cancer cell line and the CT-26 mouse colon cancer cell line were purchased from the Korean Cell Line Bank. HCT-116 and CT-26 cells were maintained in RPMI 1640 medium and Dulbecco's Modified Eagle medium, respectively. Cells were cultured at 37°C in a CO_2_ incubator (5%). The cell lines were characterized by the cell bank using DNA fingerprinting analysis, species verification test, mycoplasma contamination test, and viral contamination test. The cells were cultured at 37°C in an atmosphere containing 5% CO_2_. We made stocks for each cell line at early passages, and cultures were maintained until passage 20 (within 2 months) and then discarded.

### Evaluation of the TM4SF5 peptide vaccine as an anti-metastatic agent in a lung metastasis model of colon cancer

BALB/c mice were immunized as described above. Control mice were injected with PBS or Lipoplex(O). For the metastatic cancer animal experiments, the mice were injected intravenously (via tail veins) with CT-26 cells (1 × 10^5^) on day 30 (PBS controls, *n* = 8; colon cancer cell-injected group, *n* = 15). The body weight was measured in 2-day intervals. On day 22 after CT-26 cell injection, mice were sacrificed and the lungs were weighed.

### Examination of lung nodules

BALB/c mice were immunized and injected with CT-26 cells as described above. On day 16 (for TM4SF5 peptide vaccine group) or 20 (for PBS control group) after CT-26 cell injection, mice were sacrificed (*n* = 4 per group). The trachea was cannulated with a 20-gauge catheter and 1 mL India ink (Parker; 1:16 dilution in PBS) was injected into the lung. Lungs were extracted and destained by soaking in Fekete's solution, and the metastatic nodules were counted as previously described [33].

### Production of the mouse monoclonal antibody specific to the cyclic peptide

After intraperitoneal immunization of BALB/c mice with the TM4SF5 peptide vaccine four times at 10-day intervals, we obtained hybridoma cells producing a monoclonal antibody that specifically recognizes the TM4SF5 cyclic peptide, using established protocols in hybridoma technology [34]. The anti-TM4SF5 monoclonal antibody (IgG3), mEC2-C, was purified from the ascitic fluid using Protein A column. Purified antibody was analyzed by ELISA and the SR7500DC Reichert SPR system to investigate its binding affinity.

### Cloning of the variable heavy and light domains (Fab) of the anti-TM4SF5 monoclonal antibody

The hybridoma cells producing the anti-TM4SF5 monoclonal antibody (mEC2-C) were cultured. Total RNA was extracted from the hybridoma cells, and cDNA was generated by reverse transcription. To clone the Fab sequences of the anti-TM4SF5 monoclonal antibody, the resultant cDNA was amplified using Vent polymerase (NEB) with the following primer sets, as described previously [35]: Heavy chain primers, IGG3 : 5′-GGAAGATC TAGGGACCAAGGGATAGACAGATGG-3′, 5′MH2 : 5′-CTTCCGGAATTCSARGTNMAGCTGSAGSAGTC WGG-3′; Kappa chain primers, 3′Kc : 5′-GGTGCATGCG GATACAGTTGGTGCAGCATC-3′, 5′Mk : 5′-GGG AGCTCGAYATTGTGMTSACMCARWCTMCA-3′. The standard PCR reaction was conducted for 25 cycles and the PCR products were directly cloned using the pGEM-T Easy vector (Promega). Cloned mouse immunoglobulin gene inserts were analyzed by DNA sequencing.

### Sequence analysis and molecular modeling of variable fragment (Fv)

The immunoglobulin variable domain sequence of mEC2-C was analyzed with IgBLAST (http://www.ncbi.nlm.nih.gov/igblast/) [17]. Six CDRs determined by Kabat numbering [20] and some framework residues of the mEC2-C monoclonal antibody were grafted into the sequence for the human VH3-Vk1 subfamily (the Herceptin framework). The three-dimensional structure of both mouse and humanized EC2-C Fv amino acid sequences was simulated using the web modeling program, ROSIE [36]. This program identifies the most homologous templates for frameworks residues and CDRs of heavy and light chains; these template structures were then assembled into an optimized model. The resulting modeled structures were superimposed using Pymol software (DeLano Scientific, LLC.) rendered in a ribbon model.

### Construction and expression of humanized anti-TM4SF5 antibody

To obtain humanized IgG1 antibody with intact IgG format, VH and Vk encoding genes were synthesized (Bioneer, Korea), including restriction enzyme sites at both the 5′ and 3′ ends. The synthesized genes were then inserted into the modified pcDNA 3.4 expression vector (Invitrogen) carrying the human IgG1 constant regions (CH1-hinge-CH2-CH3) or human kappa chain constant region (CL) for mammalian cell expression in HEK 293F cells. The humanized anti-TM4SF5 monoclonal antibody (hEC2-C-2) was produced using the HEK 293F expression system, as described in previous studies [37, 38], and purified using Protein A affinity chromatography following the manufacturer's protocol after 5–7 days of cell culture. The purity of the mouse parental and humanized monoclonal antibodies was evaluated by SDS-PAGE analysis.

### Expression of recombinant human TM4SF5

The human TM4SF5 cDNA was amplified from Huh-7 mRNA by RT-PCR using the following primer set: hTM4SF5 5′ primer, 5′-CTCGAGATGTGTACGGGAAAATGTGCC-3′; hTM4 SF5 3′ primer, 5′-AAGCTTTTGTGAGGTGTGTCCTGT TTTTT-3′. The cDNA fragments were cloned into the expression vector pcDNA-3.1/Myc-His(-)B (Invitrogen). For the generation of stable cell lines expressing hTM4SF5, the HEK 293F cells (1 × 10^6^ cells/mL) were transfected with 2.5 μg/mL hTM4SF5/pcDNA and 7.5 μg/mL polyethylenimine (Polysciences), and the transfected cells were selected using 500 μg/mL G418 (Calbiochem) for 14 days. The expression of Myc-tagged hTM4SF5 was confirmed by western blot analysis with the anti-Myc-tag antibody.

### Western blot and immunoprecipitation analyses

To analyze the specificity of the monoclonal and humanized anti-TM4SF5 antibodies, cell lysates from TM4SF5-overexpressing cells were resolved on SDS-PAGE. Western blotting and immunoprecipitation assays were performed as previously described [39]. The expression of E-cadherin and β-catenin in the humanized anti-TM4SF5 antibody-treated cells was evaluated by analyzing the cell lysates by SDS-PAGE and western blotting as previously described [14].

### Confocal microscopy

To test the efficacy of recognition of TM4SF5 by the humanized anti-TM4SF5 antibody, CT-26 and HCT-116 cells were cultured on glass coverslips in 12-well plates 1 day before antibody treatment. After 3 h of treatment with DyLight 488-labeled human IgG control or DyLight 488-labeled humanized anti-TM4SF5 antibody (hEC2-C-2 Ab; 10 μg/mL), the cells were fixed, followed by staining of the nuclei with Hoechst No. 33258. The stained coverslips were mounted and scanned with an LSM 710 system (Carl Zeiss). To identify the effect of the humanized anti-TM4SF5 antibody on E-cadherin and β-catenin expression, CT-26 and HCT-116 cells were cultured and treated with the control IgG or humanized anti-TM4SF5 antibody (10 μg/mL) as above. After 3 days, E-cadherin and β-catenin expression in the cells was analyzed according to the previously described method [15].

### *In vitro* cell migration assays

Trans-well chambers with 8 μm porosity were used. The lower side of the membranes was coated with gelatin (1 mg/mL). Colon cells were suspended (1 × 10^5^ cells/mL) in serum-free medium with human IgG control or the humanized anti-TM4SF5 antibody (hEC2-C-2 Ab; 10 μg/mL) and placed on top of the trans-well chamber. RPMI medium containing 10% fetal bovine serum was placed in the lower chamber. After 24 h, cells that migrated through the porous membrane and were attached to the lower surface of the filters were fixed and stained with crystal violet for 1h. Cell numbers on at least five random fields per experimental treatment were counted under a microscope (Eclipse E-200, Nikon).

### *In vitro* wound-healing assays

CT-26 and HCT-116 cells (1 × 10^6^) were placed in a 6-well plate, cultured overnight to confluence in medium containing serum, and the monolayer was wounded with a pipette tip. PBS, human IgG control, or the humanized anti-TM4SF5 antibody (hEC2-C-2 Ab; 10 μg/mL) was added to the medium. At the indicated time points, the cells were fixed and stained with crystal violet for 30 min. The number of cells that migrated into the wounded area was counted in three wells per experimental treatment and in three wounds per well.

### Evaluation of the humanized anti-TM4SF5 antibody as an anti-metastatic agent in a lung metastasis model of colon cancer

BALB/c mice were injected intravenously (via tail veins) with 1 × 10^5^ mouse CT-26 colon cancer cells (PBS controls, *n* = 8; colon cancer cell-injected group, *n* = 36). On day 5, mice injected with cancer cells were randomly divided into three treatment groups: PBS, human IgG control, and the humanized anti-TM4SF5 antibody (hEC2-C-2) group; *n* = 12 per group. The antibodies (25 mg/kg body weight) were injected into the tail veins with the corresponding treatment twice a week. The body weight was measured in 2-day intervals. Survival of the mice was monitored until day 22.

Another experiment with the same setting was performed to examine the lungs (*n* = 12 per group). On day 19, the mice were sacrificed and lungs were weighed.

### Histology

For histopathological analyses, lung sections were prepared and stained with hematoxylin and eosin as previously described [14]. All images were examined using a microscope (Eclipse E-200, Nikon).

### Statistical analysis

Results are presented as mean ± standard deviation. Statistical significance of comparisons of differences between two samples was evaluated using Student's *t* test; differences were considered to be significant for values of *P* < 0.05. A survival analysis was performed using the Kaplan-Meier method, and the results were evaluated with the log rank test.
